# Iris Fixation for Intraocular Lens Dislocation: Relocation with Iris Suture Versus Exchange to Sutureless Iris Claw IOL

**DOI:** 10.3390/jcm13216528

**Published:** 2024-10-30

**Authors:** Carlo Bellucci, Paolo Mora, Alessandra Romano, Salvatore Antonio Tedesco, Mario Troisi, Roberto Bellucci

**Affiliations:** 1 Ophthalmology Unit, University Hospital of Parma, 43126 Parma, Italy; 2 Eye Clinic, Department of Neurosciences, Reproductive and Odontomastological Sciences, University of Naples Federico II, 80138 Naples, Italy; troisi165@gmail.com; 3 Ophthalmology Clinic, Sant’ Anna Hospital, 25127 Brescia, Italy

**Keywords:** Siepser slipknot, iris claw IOL, dislocated IOL, iris fixation, IOL repositioning, IOL exchange

## Abstract

**Background/Objectives**: To compare the clinical outcome of suture and sutureless iris fixation techniques for dislocated intraocular lenses (IOLs). **Methods**: Retrospective cohort study including patients who underwent surgery for late IOL dislocation over a 10-year period. IOL repositioning was achieved either by suturing the original IOL to the iris using the Siepser slipknot technique or by replacing it with a retropupillary sutureless iris claw IOL. Data collected during surgery included the type of dislocation, the need for anterior or posterior vitrectomy, duration of surgery, and intraoperative complications. Six months after surgery we assessed the eye and iris anatomy; refraction, astigmatism, and visual acuity; endothelial cell damage; and rate of postoperative complications. **Results**: Included in the study were 60 patients: 32 underwent IOL relocation and 28 underwent IOL exchange. Pseudoexfoliation (43.7% and 39.3%) and retinal surgery (34.4% and 28.6%) were identified as the main possible causes for IOL dislocation. The mean duration of the surgery was 62.9 ± 14.9 min for the Relocation group, and was 42.7 ± 11.4 min for the Exchange group (*p* < 0.001), with similar low intraoperative complication rates (6.25% and 7.14%, respectively). The studied parameters showed no differences between the two groups postoperatively, except for corneal astigmatism which was 1.31 ± 0.45 D in the Relocation group and was 1.89 ± 0.86 D in the Exchange group (*p* < 0.001). **Conclusions**: Both suture and sutureless iris fixation techniques for dislocated IOLs yielded similarly favorable outcomes in this study. IOL relocation resulted in less postoperative astigmatism, while IOL exchange offered the advantage of shorter surgical time.

## 1. Introduction

Although the modern design of recent intraocular lenses (IOLs) has allowed them to preserve their function and position for many decades, some IOLs may displace at various times after surgery. Usually, the displaced IOL is still contained within the capsular bag, which is also displaced, but sometimes a part of or the whole IOL can be found outside the capsular bag. In these cases, a malposition occurring at surgery or soon after surgery is suspected. The most common causes of capsular bag/IOL dislocation are zonular weakness (for example, in those who have Marfan, pseudoexfoliation syndromes or hyperhomocysteinemia), blunt trauma, surgical trauma, or a combination of these can be the culprit of bag displacement [1].

Several surgical techniques are available to relocate a dislocated IOL, which can be classified in suture/sutureless scleral fixation and suture/sutureless iris fixation [2]. Suture transscleral posterior-chamber IOL (PC-IOL) fixation techniques can only be applied to selected IOLs with suitable haptics. They require the use of a suture knot to be buried inside the sclera, which may cause complications such as knot exposure or suture breakage with a second IOL dislocation, along with the need for surgical manipulation of the sclera [3,4]. Sutureless intrascleral PC-IOL fixation techniques have been developed to overcome these complications by creating a scleral tunnel with small needles or with vitreous retinal knifes [5], through which the IOL haptics or IOL supporting Prolene may be fixed to the sclera [6,7]. This results in being less traumatic on ocular tissues than suture transscleral fixation but cannot be applied to every IOL and condition.

As scleral fixation is gaining more popularity nowadays, iris fixation remains a viable option for surgeons. Iris fixation is usually performed by means of the Siepser slipknot technique, which employes the use of a 10.0 prolene suture to suture the two haptics of the subluxated IOL to the iris. This technique was developed with the rationale of being less traumatic than any transcleral surgery and less time-consuming, along with reducing to the minimum the intra and postoperative risks [8]. However, iris fixation with the Siepser suture requires a thin and undamaged haptic and cannot be applied to every IOL and condition [9,10].

When the dislocated IOL cannot be re-used because of the shape of the haptics, some optics or haptics damage, or because of excessive lens material proliferation, the decision to exchange it for a new IOL is considered. In these cases, the most frequent choice is the implantation of an iris–claw intraocular lens (IC-IOL) [2]. Although these IOLs were originally designed to be placed in the anterior chamber (pre-pupillary), posterior chamber fixation (retro-pupillary) has become increasingly common and now it is the method preferred by most surgeons [11]. Moreover, the IC-IOLs implant has been recently proven to be associated with a lower postoperative refractive error when compared to the sutureless Yamane Double-Needle technique for scleral IOL fixation [12].

Although the literature includes numerous studies comparing suture and sutureless techniques for the scleral fixation of dislocated IOLs, less attention has been dedicated to iris fixation, with or without IOL exchange. In particular, we could not find any paper comparing dislocated IOL relocation with the Siepser sliding knot suture with dislocated IOL exchange for an iris–claw IOL posteriorly fixed to the iris. Since these have been our preferred techniques over the last 10 years, we decided to collect and present our experience in this paper.

## 2. Materials and Methods

This retrospective cohort study was conducted in accordance with the tenets of the Declaration of Helsinki and approved by the local Ethical Committee. The included patients were recruited from the paper and electronic records of patients who underwent surgery to manage late IOL dislocation over a 10-year period from January 2014 to December 2023. All the surgeries were performed by the same surgeon.

Late IOL dislocation was defined as any case requiring IOL repositioning surgery in which the initial IOL position had been noted as good and stable for 6 months after cataract surgery. No patient was excluded, regardless of the status of the capsular bag, and to ensure the study to be a true representation of the real-world setting. Eyes with complete IOL luxation into the vitreous cavity were included. Any IOL type and any previous additional surgery like glaucoma or posterior vitrectomy was accepted, provided the lens had been implanted into an undamaged capsular bag at the time of the original cataract surgery. We decided not to include eyes with a torn posterior capsule at cataract surgery and eyes with a traumatic cataract to make our group more homogeneous.

Indications for the relocation surgery was the presence of a luxated or subluxated IOL in a seeing eye. The status of the contralateral eye was not considered of relevance for the surgical decision. Old age was not a contra-indication. No general condition was considered as a contra-indication, with the exception of dementia in patients not suitable for general anesthesia. A total of 135 patients in whom the IOL was relocated were identified. Of those, 24 cases were early dislocation because of a torn posterior capsule or an unsatisfactory implantation recorded at the original surgery, 21 were original traumatic cataract cases, and 30 had undergone different types of scleral fixation of the IOL: all these eyes were excluded. A total of 60 eyes underwent either iris suturing of the haptics of the dislocated IOL (Siepser technique) or an IOL exchange to a retropupillary iris–claw IOL and were included in this study. This paper includes all the eyes that were operated on with the described techniques in the considered period.

### 2.1. Surgical Technique

Every case was analyzed individually to prepare the relevant surgical plan. The first option was to try to relocate the IOL to minimize the surgical trauma and to avoid larger corneoscleral incisions that might result in an increase in astigmatism. The decision to exchange the luxated IOL was made preoperatively when the haptics were plate-haptic, closed loop, thick, or if the power had been originally inadequate. IOLs with thin open haptics, either three-piece or single-piece, were planned to be exchanged only if discovered damaged during surgery, or if the tissue proliferation inside the capsular bag was considered too excessive to be removed safely.

Mydriasis was obtained with 0.5% tropicamide/10% phenylephrine eye drops. In all cases, local peribulbar anesthesia with 6–8 mL of 2% mepivacaine was performed. Two injections were made, the first delivering 4–5 mL into the infero-temporal peribulbar space and the second delivering 2–3 mL into the superior rectus zone. Thereafter, a 25 G transconjunctival irrigation trocar was placed 3.5 mm from the limbus in the inferotemporal eyeball quadrant, with the purpose of activating irrigation if needed to help the anterior vitrectomy and to prevent the IOL from falling into the vitreous chamber, or to serve as irrigation when a posterior vitrectomy was needed. For the purpose of this study, anterior vitrectomy is defined as any vitrectomy performed through corneoscleral incisions, and posterior vitrectomy is defined as any vitrectomy performed through the pars plana using trocars and the relevant observation system. At the end of the surgery, 0.2 mL of 1% cefuroxime was injected in all cases. Postoperatively, all the eyes received a combination of steroid/antibiotic eye drops q.i.d. and an NSAID agent b.i.d. for 2–4 weeks according to the amount of inflammation.

### 2.2. IOL Relocation

Two or three 1 mm limbal corneal incisions were prepared in convenient meridians according to IOL loop position. The anterior chamber was filled with adhesive viscoelastic material. The IOL was then brought into the anterior chamber with one of the following maneuvers; these were frequently finalized by retinal forceps introduced through a limbal incision: (1) the IOLs horizontally dislocated behind the iris were pushed upwards into the anterior chamber by a cataract manipulator first slipped behind the IOL optics and then lifted; (2) the IOLs vertically positioned to the iris plane and with one loop attached to the zonula were pushed horizontally and moved into the anterior chamber by an STC-6 straight needle (Ethicon sutures) introduced through the pars plana close to the attachment and the IOL optics; (3) the IOLs completely dislocated within the vitreous required a full posterior 25 G vitrectomy, sometimes using perfuorocarbon fluids to protect the posterior retina, and were moved into the anterior chamber either by grasping one loop or the surrounding capsule with retinal forceps or by suction using a Charles cannula. Anterior vitrectomy using a posterior vitreous cutter was performed whenever needed, aided by triamcinolone to visualize the vitreous.

Once in the anterior chamber, using the vitrector and scissors, the IOL was cleaned as much as possible from lens and capsule material that could interfere with the surgery or with the postoperative vision. The pupil was constricted with 1% acethylcholine. One loop of the IOL was placed through the pupil behind the iris and sutured to the iris in the mid periphery with a 10/0 prolene suture attached to an STC-6 straight needle using the Siepser sliding knot technique. Thereafter, the second loop was dragged behind the iris and sutured in the same way. Care was taken to avoid pupil ovalization, although the exact position of the sutures was sometimes imprecise due to residual mydriasis or to poor corneal transparency. The posterior irrigation was activated at intervals to maintain the eye shape. The anterior chamber was checked by Triamcinolone injection and any vitreous remnant was removed. Finally, the viscoelastic substance was washed with separate irrigation and aspiration cannulas, and 1% cefuroxime was injected. No iridectomy was performed. The incisions were closed by hydration, and no corneoscleral suture was applied.

### 2.3. IOL Exchange

After moving the IOL into the anterior chamber and after achieving miosis, a 6 mm frown corneoscleral incision was prepared at the superior limbus, with two 1 mm incisions at both sides to be used for iris fixation of the new IOL. The luxated IOL with all the bag remnants was removed and the new iris–claw IOL (Artisan aphakia, Ophtec, Groningen, The Netherlands) was introduced upside-down into the anterior chamber and left in front of the iris. The IOL was then rotated 90° horizontally and grasped with the dedicated forceps. The first claw was moved behind the iris and fixated to the posterior peripheral iris with the dedicated enclavator needle which was inserted through the relevant 1 mm incision. The IOL was then slipped behind the iris and the second claw fixated to it in the same way. Care was taken to avoid pupil ovalization, although the exact position of the enclavation was sometimes imprecise due to residual mydriasis or to poor transparency of the peripheral cornea. The posterior irrigation was activated at intervals to maintain the eye shape. The anterior chamber was checked, and any vitreous remnants were removed. The 6 mm incision was closed with a running 10/0 nylon suture, designed to remain as long as possible to counteract the development of against-the-incision astigmatism. Finally, the viscoelastic substance was washed with double irrigation and aspiration cannulas, and 1% cefuroxime was injected. No iridectomy was performed. The power of the new iris–claw IOL was selected using the Barrett Universal II formula with a constant of 116.9.

### 2.4. Considered Parameters

The records of these patients were used to collect the study data: basic demographics; eye condition at the original surgery when available: presence of pseudoexfoliation (PEX), state of the cataract and of the pupil, ocular comorbidity and the use of drugs known to cause floppy iris.

The examination performed prior to the relocation/exchange surgery provided additional data: time from the original surgery to the onset of visual symptoms, additional surgery in the interval, corneal transparency, endothelial cell count, previous Nd: YAG laser posterior capsulotomy, corrected distance visual acuity (CDVA), and intraocular pressure (IOP). The figures of keratometry, axial length, and pupil diameter were assessed by ocular biometry (IOL Master 500, Zeiss, Oberkochen, Germany). The status of the retina was assessed with ophthalmoscopy and with Fourier–Domain or Swept-Source Ocular Coherence Tomography (OCT). IOL dislocation was termed as grade 1 when the IOL optics were within the optical axis, grade 2 when one haptic was visible in the pupillary zone, grade 3 when the IOL was attached to a thin inferior strand of zonule fibers, and grade 4 when the IOL was completely in the vitreous or over the posterior pole.

During the surgery, we recorded the intra-operative difficulties and complications: the status of the pupil and of the iris, the position of the IOL when the patient was in the supine position, the status of the iris at the end of the surgery, and the total surgical time.

In the postoperative period, we recorded the need for secondary procedures like early IOL re-fixation, correction of iris malposition, and early suture complication. Patients were followed up every week for one month in particular to check intraocular pressure, and every 2 months thereafter principally looking for macular complications like cystoid macular edema. The 6-month ophthalmic visit was considered final. Patients underwent a full ophthalmic evaluation, with a particular emphasis on the endothelial cell count, corneal astigmatism, and pupil ovalization. The status of the pupil was graded from 0 to 3 according to the difference between the maximum and minimum pupil diameter: grade 0 meant <1 mm, grade 1 meant 1 mm to 2 mm, grade 2 meant 2 mm to 3 mm, grade 3 meant >3 mm. It should be noted that at this visit, all the sutures in the Exchange group were in place.

### 2.5. Data Analysis

Visual acuity was generally recorded in Snellen notation, and it was converted to LogMAR for arithmetic procedures. Statistical analysis was performed using SPSS for Mac software (version 25, SPSS, Inc., Chicago, IL, USA). For the comparison between the two groups, the Pearson’s chi-square (χ^2^) test was used for the categorical data and independent samples *t*-test for continuous parameters, after checking for normal distribution with the Shapiro–Wilk test. If the number of cases was less than five in each group, the Fisher’s exact test was used. For within-group data comparison before and after surgery, a paired samples *t*-test was used. A *p*-value of less than 0.05 was considered statistically significant. Results are presented as mean with a standard deviation, median, and range or number (percentage) as appropriate.

## 3. Results

This study includes 60 eyes of 60 patients, of which 32 underwent IOL relocation and 28 underwent IOL exchange. The current follow-up is 8–127 months. The characteristics of the included eyes are collected in Table 1. The only difference found between groups was related with age, which was younger for the IOL exchange group. The pupil diameter at the original cataract surgery was only available for 18 eyes in the Relocation group (7.7 ± 0.9 mm) and 6 eyes in the Exchange group (6.9 ± 0.9 mm) without showing any significant difference (*p* = 0.095).

As for the possible causes of the IOL luxation, we were able to identify previous posterior vitrectomy in 11 eyes (Relocation) and in 8 eyes (Exchange), which had been performed because of retinal detachment in 5 eyes (Relocation) and in 4 eyes (Exchange). Pseudoexfoliation was observed in 14 eyes and 11 eyes, respectively. All the patients with glaucoma were using topical medications, and their IOP was within normal values. In four eyes (Relocation) and in eight eyes (Exchange), no possible cause of IOL luxation was identified: in these eyes, we can postulate either an unknown zonular disease or the late consequences of excessive surgical trauma.

The type of IOL dislocation considered in this study was assessed at surgery, with the patient supine; this is because some IOLs apparently located in the iris plane assume a vertical position under the operating microscope (Table 2). Out of the IOLs lying horizontal with the patient supine, 11 were centered and 11 decentered in the Relocation group, and six were centered and four were decentered in the Exchange group. A total of 32 IOLs were three-piece: of those, six had to be replaced either because of haptics damage or because of excessive intracapsular proliferation. A total of 28 IOLs were single piece: six of those had thin haptics and were selected for relocation.

No retinal detachment was observed in any eye at surgery. Other retinal morbidities included optic nerve head excavation (three and four), myopic retinal atrophic zones (four and one), myopic retinoschisis (one and zero), different types of macular degeneration (eight and three), epiretinal membranes (three and two), and previous central retinal vein occlusion (zero and one). These morbidities were considered not related to the purpose of this study and were not analyzed in further detail.

**Figure 1:**
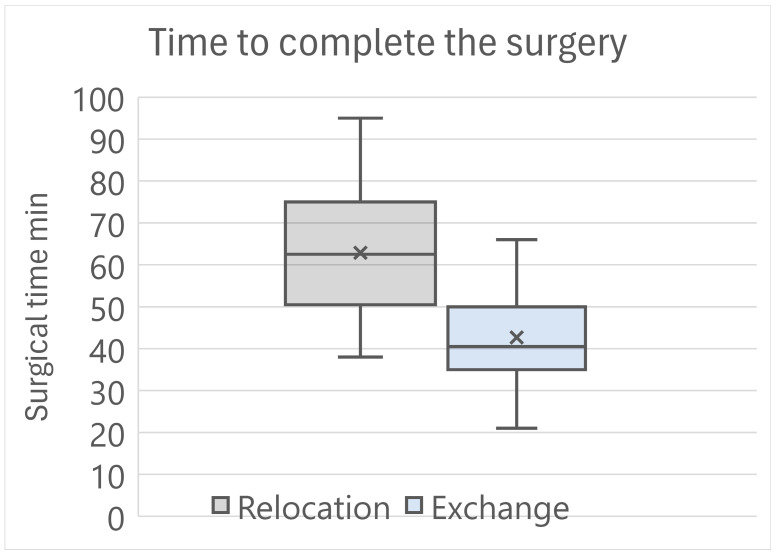
Time required to complete the surgery with the two adopted techniques. The surgical time was 62.9 ± 14.9 min in the Relocation group and was 42.7 ± 11.4 min in the Exchange group. The difference is statistically significant (*p* < 0.001).

All the surgeries were performed by two of the authors (PM and RB). At surgery, the number of eyes requiring no vitrectomy, anterior vitrectomy, and posterior vitrectomy were 5/12/15 in the Relocation group, and were 1/8/19 in the Exchange group (*p* = 0.158) (Table 3). The time to complete the surgical procedure was 62.9 ± 14.9 min for the Relocation group, and was 42.7 ± 11.4 min for the Exchange group, with a highly statistically significant difference (*p* < 0.001) (Figure 1).

Intraoperative complications occurred with both techniques. In the Relocation group, one posterior hemorrhage that eventually led to vision loss occurred in one highly myopic eye where the IOL had dislocated over the posterior pole. In addition, one iris bleeding that was eventually controlled, with no consequences, also occurred. In the Exchange group. we had difficulties during IOL enclavation in two eyes, in one of which the pupil border had slipped into the claw and was then set free with difficulty. No specific intraocular inflammation was observed in these eyes after surgery.

**Figure 2:**
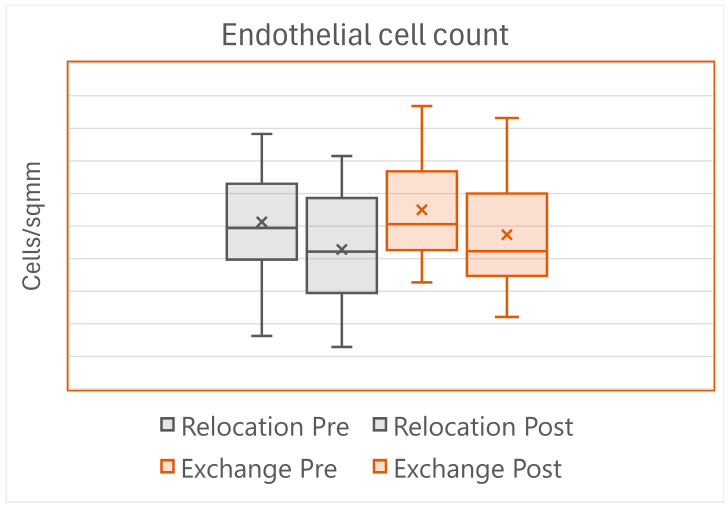
Box plots reporting the central endothelial cell counts before and 6 months after surgery in the study eyes. In the Relocation group, the preoperative count was 2026 ± 309 cells/mm^2^, and the postoperative count was 1856 ± 325 cells/mm^2^. In the Exchange group, the preoperative count was 2100 ± 334 cells/mm^2^, and the postoperative count was 1947 ± 337 cells/mm^2^. The differences between groups were not statistically significant.

Postoperatively, corneal edema lasting more than 5 days was found in seven eyes in the Relocation group, and in the Exchange group in two eyes (*p* = 0.155), but all the corneas were clear at the sixth-month visit. One IOL per group had to be re-fixed to the iris. The endothelial cell loss showed great variation across eyes, reflecting the wide variation in the surgical maneuvers and time (Figure 2).

**Figure 3:**
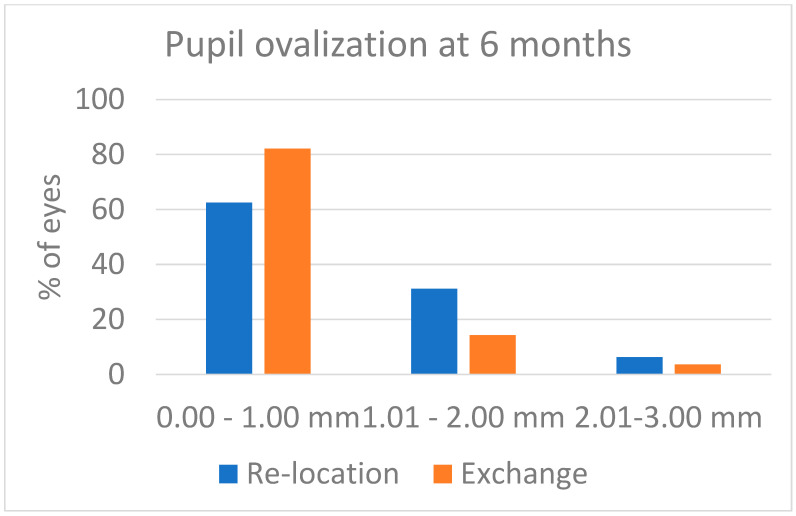
Pupil ovalization observed 6 months after surgery, expressed as the difference between the minimum and the maximum corneal diameter. The difference between groups was not significant (*p* = 0.239).

**Figure 4:**
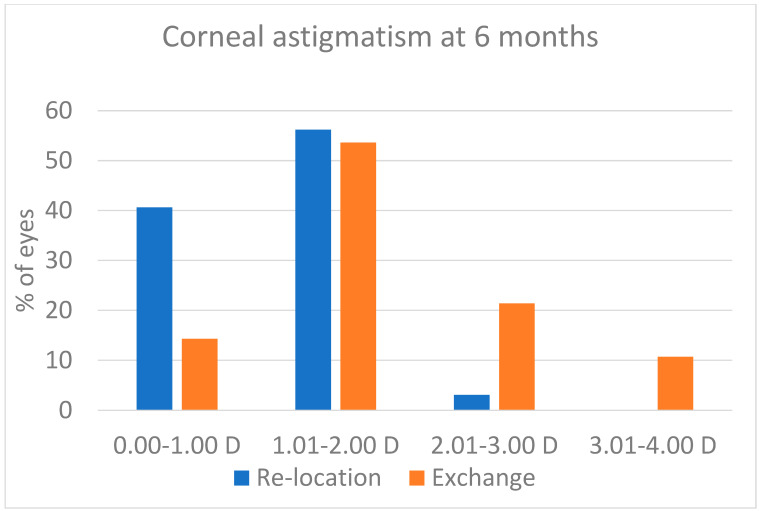
Corneal astigmatism at the 6-month visit. The difference between groups was statistically significant (*p* = 0.001).

Some pupil ovalization developed in both groups: it was evident after surgery, and it decreased over the first weeks after surgery. At the 6-month visit, the figures were those reported in Figure 3. Despite the values being a little higher in the Relocation group, the difference was not statistically significant (*p* = 0.239). The postoperative corneal astigmatism as recorded 6 months after surgery was 1.31 ± 0.45 D in the Relocation group and was 1.89 ± 0.86 D in the Exchange group (*p* = 0.001). The distribution of postoperative corneal astigmatism is shown in Figure 4. As compared with the preoperative values, the corneal astigmatism increased by 0.15 D in the Relocation group (11.5% of the preoperative value), and by 0.56 D in the Exchange group (42.1% of the preoperative value). This difference between groups is statistically significant (*p* = 0.024).

The refractive spherical equivalent of the 6-month visit was −0.93 ± 0.98 D in the Relocation group and was −0.82 ± 1.32 D in the Exchange group (*p* = 0.879). The clinical astigmatism was 1.14 ± 0.52 D in the Relocation group and was 1.77 ± 0.95 D in the Exchange group (*p* = 0.001), probably influenced by some tilt and decentration of the IOLs. Corrected distance visual acuity was 0.27 ± 0.12 LogMAR and 0.33 ± 0.26 LogMAR, respectively. The small difference was not statistically significant (*p* = 0.182).

Three eyes per group developed cystoid macular edema. One eye with high myopia in the Relocation group lost vision as the result of macular atrophy developing after massive intraoperative posterior hemorrhage occurring at the end of the anterior surgery. One eye in the Exchange group suffered a retinal detachment 5 months after surgery, which was successfully repaired by pars plana vitrectomy and gas tamponade.

## 4. Discussion

Like most studies on IOL dislocation treatment, our study is observational in nature, as the particular features of the involved eyes make it difficult to randomize them to a specific treatment [13]. Posterior chamber intraocular lenses originally implanted into the capsular bag can dislocate at various times after surgery. They usually dislocate with the capsular bag, but sometimes they dislocate out of the bag, because of a much too large capsulorhexis or of later vitreoretinal surgery [14]. The mean age of the patients found with dislocated IOLs varies across papers, from a minimum of 63 years [15] to a maximum of 84 years [16], with a tendency to be lower than the age of control patients operated on for cataracts at the same time [17]. The mean age of our patients was 79.63 ± 7.22 years, with a median of 81 years. It should be noted that the mean age of the Relocation group was higher than that of the Exchange group by 7.4 years, probably reflecting the progressive abandon of the three-piece IOLs in our area. There were no differences between our two groups of patients as for laterality (right/left eye) and for gender (male/female).

The time interval from the cataract surgery to the diagnosis of IOL dislocation varies widely: it was reported to be between 3.4 years and 12.5 years in the review published by Kristianslund et al. [13], and a mean time of 8.1 ± 4.0 years was reported by Riedl et al. in the 150 patients analyzed in 2023 [18]. Our patients experienced IOL dislocation 6.78 ± 3.03 years after the original cataract surgery, with a median interval of 73.5 months, with no difference between the two study groups.

The probable cause of the IOL dislocation (Table 1) was identified in 48 patients of the cohort (80.0%), in 28 patients of the Relocation group (87.5%), and in 20 patients of the Exchange group (71.4%), with no difference between groups. Pseudoexfoliation, with or without glaucoma, was found in 41.7% of our eyes, in line with the 20–68% figures in the Kristianslund et al. paper [13] and with the 30.7% figure in the Riedl et.al. paper [18], confirming this is the main cause for late IOL dislocation. Previous retinal surgery was the probable cause of IOL dislocation in 31.7% of our patients, also in line with the 33–40% figures [13] and with the 30.7% figure [18] found in the literature, again with no difference between groups.

Out of the dislocated IOLs, 32 were three-piece and 28 were single-piece. In the Relocation group, most IOLs were three-piece (26 vs. 6), while in the Exchange group most IOLs were single-piece (22 vs. 6), with a statistically significant difference between groups. This depends on our choice not to relocate by iris suture single-piece IOLs with thick haptics, despite that there are reports about successful scleral suturing of these IOLs [19].

The best corrected visual acuity we found in preoperatively was 0.47 ± 0.23 LogMAR (Median: 0.44 LogMAR), with no difference between groups (Table 1). Corneal astigmatism as measured by corneal topography was also similar: an important finding as the amount of corneal astigmatism is one of the main outcome parameters of this study. Intraocular pressure, keratometry, and axial length also showed no difference between groups in preoperatively (Table 1).

The surgical technique we prefer for three-piece IOL relocation is iris suturing of the loops with the Siepser sliding knot technique [9]. We switched to this approach from the scleral fixation we employed years ago [20] to avoid bleeding from the ciliary body, and to overtake the lack of precision in the placement of the scleral sutures, sometimes leading to IOL tilting requiring further surgery. We have continued with this approach even in despite of more recent techniques [2] because iris fixation can be applied to any three-piece or thin haptics IOL, it does not require scleral manipulation with the risk of suture or haptic exposure, and does not need specific training outside of our expertise. Similar considerations apply to IOL exchange: posterior iris fixation of the iris–claw Artisan IOL—an IOL we are familiar with since 1986—has been considered the quickest and safest way to implant an IOL in an aphakic eye [2].

At surgery, we noted a difference in the pupil diameter between the two groups. The patients who underwent IOL exchange had a smaller pupil diameter, probably as a consequence of the more frequent previous vitreoretinal surgery. There was also an obvious difference in the difficulty of the maneuvers according to the type of IOL luxation. The frequent need for posterior vitrectomy raises the concern about IOL dislocation surgery, which should be addressed only by surgeons who are experts in posterior segment surgery, or in co-operation with them. As for the type of surgery, the number of intraoperative complications we had was small: one hemorrhage in the vitreous chamber, that eventually led to vision loss, and one iris bleeding that was well controlled intraoperatively. We did not consider the complexity and the need for repetition of the maneuvers to secure the IOL to the iris as a complication. However, these difficulties are reflected in the time required to complete the surgery. Figure 1 clearly points out that IOL exchange required by far less surgical time than IOL relocation, because it did not involve the need to control the IOL, to keep it safe from surgical damage, or to clean the surrounding capsular bag remnants. In addition, the Siepser sliding knot suture takes longer to apply than iris enclavation of the Artisan IOL. The difference between the two techniques of iris fixation is both statistically and clinically important and suggests favoring IOL exchange over IOL relocation in every eye with IOL dislocation where iris fixation is selected.

Postoperative complications were similar between the two techniques, with one IOL per group that had to be re-fixed to the iris. The anatomic and the visual outcome were also similar: the endothelial cell loss was the same despite the longer surgical time of the Relocation group (Figure 2), and the visual acuity at the 6-month visit showed some increase as compared with preoperatively, with no difference between groups.

Postoperative corneal astigmatism was one of the main outcome measures of this investigation. We are aware that the 6-month visit may be adequate for the Relocation group and may be adequate at a lesser extent to evaluate the long-term outcome of sutured 6 mm frown corneoscleral incisions that were used in the Exchange group, with the sutures still in place [21]. However, the patients are usually interested in the short- and medium-term outcome of their surgery, so the 6-month astigmatism has clinical value even ahead of later changes and is a good parameter to compare the two techniques. The results we obtained clearly point to the superiority of the relocation strategy in this regard. While the two groups had similar corneal astigmatism in preoperatively, the eyes in the Relocation group had a lower increase in the corneal astigmatism with surgery (0.15 D vs. 0.56 D, *p* = 0.024), and lower corneal astigmatism at the 6-month visit (1.31 ± 0.45 vs. 1.89 ± 0.86 D, *p* = 0.001). In one eye of this group, and in nine eyes in the Exchange group, the corneal astigmatism was higher than 2.0 D.

A common problem of iris fixation is the pupil dynamics [22,23]. In our patients, we did not check the pupil dynamics, but only looked for pupil ovalization by measuring the difference between the largest and the smallest pupil diameter. The results in Figure 3 indicate there was no difference between the two techniques, i.e., iris suturing of IOL haptics and iris enclavation of lobster claw haptics had the same impact on pupil shape as measured 6 months after surgery.

Many papers have been published comparing different techniques to address IOL dislocation, although we could not find in the literature any paper comparing the two techniques of iris fixation that we are comparing here: loop suture and loop enclavation. In their review published in 2020, Shen et al. compared eight different techniques of IOL implantation in the absence of capsular support and concluded that the evidence reviewed showed no superiority of any single IOL implantation technique [24]. The various techniques seemed to have equivalent visual acuity outcomes and safety profiles. According to the collected evidence, each technique has its own profile of inherent risk of postoperative complications, and surgeons are encouraged to educate patients on the importance of close and long-term follow-ups as a result of the uncertain nature of these techniques.

The possible disadvantages of the re-use of dislocated IOLs were addressed by Baba et al. [25]. In their small series, three out of six re-used IOLs required further surgery, and therefore they suggest exchange vs. relocation. It was our decision to inspect every IOL looking for damage, and to exchange any damaged IOL. Therefore, in this series, only one patient per group required further surgery, and no patient in the Relocation group required IOL exchange after the relocation surgery. The equal safety and effectiveness of IOL relocation vs. IOL exchange found by us is a further confirmation of the result of the meta-analysis performed by Yang et al. [26]. However, in their report, Yang et al. did not consider both surgical time and postoperative astigmatism as important endpoints.

Many papers report the comparison between IOL relocation with scleral fixation and IOL exchange for the Artisan iris–claw IOL. The review published by Lau [27] points out the similar refractive and visual results, however with more complications in the scleral fixation group. More recent studies also found similar refractive and visual outcomes between the two techniques, with astigmatism after 6 months which was similar [28] or slightly higher in the Exchange group [29]. Interestingly, the shorter surgical time required by IOL exchange vs. IOL relocation has been addressed only recently [29].

The comparison of IOL relocation with scleral fixation using the Yamane technique and of IOL exchange with the Artisan iris fixation is more recent. Kelkar et al. [30] found similar efficacy and safety, but with pupil ovalization in 20% of the Artisan eyes. In 2024, Guerin et al. [12] reported about 116 patients in whom secondary IOL implantation had been performed: in 50%, the Yamane technique was adopted, and in 50%, the Artisan iris fixated IOL was implanted. They conclude a similar safety of the two techniques, with better refractive precision in the Artisan group. Despite that, they do not mention surgical time or pupil ovalization as outcome measures; this paper is interesting because it demonstrates that old technology (the Artisan IOL was designed almost 50 years ago) can withstand the comparison with a very recent technique.

In the introduction of this paper, we mentioned the reasons why we still prefer iris fixation over other techniques to fix IOLs in the absence of capsular support. The results of our study regarding surgery for IOL dislocation comparing IOL relocation with iris suture of the haptics and IOL exchange with Artisan IOL implantation favor IOL exchange for surgical time, and favor IOL relocation for postoperative astigmatism. Considering the age and the ocular condition of many of our patients with a dislocated IOL, we think IOL exchange should be preferred.

As with most studies dealing with IOL dislocation, this study is retrospective and observational in nature and as a consequence some interesting parameters like IOL tilt and centration were not available for all the patients and were not included in this study. In addition, yearly follow-up was not available for all the patients, and therefore late pupil changes occurring after the sixth month visit also were not included. The power of the study could not be defined to set the sample size, and the number of the included patients might not be ideal to draw the conclusions for all the studied parameters. Despite those limitations, this is the first study comparing two methods (suture and sutureless) for iris fixation of IOLs in aphakic patients, and we thought it important to share our experience in the field.

## Figures and Tables

**Table 1:** Characteristics of the included patients/eyes. CDVA was obtained with aphakic correction in some eyes.

Parameter	IOL Relocation		IOL Exchange		*p*
	N	(%)	N	(%)	
Patients	32		28		
Male/Female	20/12	(62.5/37.5)	16/12	(57.1/42.9)	0.793
Age (yr, mean ± SD)	83.1 ± 5.0		75.7 ± 7.5		<0.001
Range	73–92		54–92		
Median	82		76		
Right/Left eye	18/14	(56.2/43.8)	16/12	(57.1/42.9)	0.801
Eyes with co-morbidities	28	(87.5)	20	(71.4)	0.195
Previous Retinal Surgery	11	(34.4)	8	(28.6)	0.782
Glaucoma	6	(18.8)	4	(14.3)	0.737
Pseudoexfoliation	14	(43.7)	11	(39.3)	0.796
Eyes with no co-morbidity	4	(12.5)	8	(28.6)	0.195
Floppy iris	4	(12.5)	4	(14.3)	1
Time to luxation (mo, mean ± SD)	81 ± 32		81 ± 41		0.936
Range	32–176		24–202		
Median	75		71		
Three-piece IOL/One-piece IOL	23/9	(71.9/28.1)	8/20	(28.6/71.4)	0.002
Previous YAG	28	(87.5)	26	(92.8)	0.675
CDVA (logMAR, mean ± SD)	0.45 ± 0.17		0.49 ± 0.28		0.520
Range	0.80–0.14		0.10–1.00		
Median	0.44		0.42		
IOP (mmHg, mean ± SD)	18.8 ± 2.2		16.7 ± 5.2		0.046
Range	14–23		8–28		
Median	18		16		
Corneal astigmatism (D, mean ± SD)	1.16 ± 0.65		1.33 ± 0.71		0.3371
Range	0.18–2.26		0.15–3.25		
Median	1.04		1.11		
Endothelial cell count (n/mm^2^, mean ± SD)	2026 ± 309		2100 ± 334		0.615
Range	1325–2566		1654–2655		
Median	1988		2012		
Axial length (mm, mean ± SD)	24.22 ± 2.00		23.77 ± 1.53		0.565
Range	22.7–29.8		22.6–29.5		
Median	23.35		23.16		
Pupil diameter (mm, mean ± SD)	6.64 ± 0.81		5.60 ± 1.15		<0.001
Range	5.5–8.0		4.0–7.0		
Median	7.0		5.50		
IOL Power (D, mean ± SD)	Not Available		+18.66 ± 2.53		

IOL: intraocular lens; N: amount of the tested parameter; yr: years; mo: months; SD: standard deviation; YAG: laser capsulotomy; CDVA: corrected-distance visual acuity; IOP: intraocular pressure; D: diopters.

**Table 2:** Type of IOL luxation. The Fisher exact test is significant (*p* = 0.037).

Parameter	IOL Relocation		IOL Exchange	
	N	(%)	N	(%)
Horizontal	22	(34.4)	10	(35.7)
Centered (Grade 1)	11		6	
Decentered (Grade 2)	11		4	
Vertical (Grade 3)	7	(21.8)	12	(42.9)
Posterior pole (Grade 4)	3	(9.4)	6	(21.4)

IOL: intraocular lens; N: amount of the tested parameter.

**Table 3:** Type of vitrectomy, surgical time, and endothelial cell loss in the two study groups.

	IOL Relocation	IOL Exchange	*p*
Vitrectomy			
No	5	1	
Anterior	12	8	0.158
Posterior	15	19	
Surgical time (min, mean ± SD)	62.9 ± 14.9	42.7 ± 11.4	<0.001
Range	39–95	21–66	
Median	62	40	
Endothelial cell loss (%, mean ± SD)	8.43 ± 7.01	7.36 ± 4.87	0.415
Range	0.13–25.74	0.81–20.77	
Median	5.34	6.77	
Intra-operative complication	2	2	0.890
Vitreous hemorrhage	1	0	
Iris bleeding	1	0	
Difficult enclavation	0	2	

IOL: intraocular lens; min: minutes, SD: standard deviation.

## Data Availability

The data presented in this study are available on request from the corresponding author.
